# Aging, immunosenescence and membrane rafts: the lipid connection

**DOI:** 10.1186/2046-2395-1-6

**Published:** 2012-10-04

**Authors:** Tamas Fulop, Aurélie Le Page, Hugo Garneau, Naheed Azimi, Sarra Baehl, Gilles Dupuis, Graham Pawelec, Anis Larbi

**Affiliations:** 1 Department of Medicine, Research Center on Aging, Graduate Program in Immunology, Faculty of Medicine and Health Sciences, University of Sherbrooke, 3001 12th Avenue North, Sherbrooke, Qc, J1H 5N4, Canada; 2 Department of Biochemistry, Graduate Program in Immunology, Faculty of Medicine and Health Sciences, University of Sherbrooke, 3001 12th Avenue North, Sherbrooke, Qc, J1H 5N4, Canada; 3 Center for Medical Research, Tübingen Aging and Tumor Immunology Group, University of Tübingen, Waldhörnlestrasse 22, Tübingen, D-72072, Germany; 4 Singapore Immunology Network (SIgN), Immunos Building/Biopolis, Agency for Science Technology and Research (A*STAR), 8A Biomedical Grove, Singapore, 138648, Singapore; 5 Research Center on Aging, University of Sherbrooke, 1036, rue Belvedere Sud, Sherbrooke, Qc, J1H 4C4, Canada

**Keywords:** Cholesterol, Immune synapse, Immunosenescence, Lipid rafts, Signal transduction, T cells

## Abstract

The decreased efficiency of immune responses in older people is partly a consequence of alterations in T lymphocyte functions caused by modifications in the early events of signal transduction. Several alterations in the signaling pathways of T lymphocytes have been described in older humans and animals. A unifying cause could be modifications in the physicochemical properties of the plasma membrane resulting from changes in its lipid composition and the distribution and function of lipid rafts (LR). The latter serve to assemble the initial components of the signaling cascade. Accumulating data suggest that the function of plasma membrane LR is altered with aging; we hypothesize that this would significantly contribute to immune dysregulation. The role of aging and cholesterol in LR functions in T lymphocytes is reviewed and discussed here.

## Review

Aging is a complex and multifactorial process. One of the outcomes of aging is the appearance of numerous diseases that are caused by alterations in different physiological systems. These changes appear to occur progressively throughout life and become more pronounced with age. Alterations in the homeostatic status of the body with aging decrease its capacity to cope with daily challenges, resulting in an increased, overall level of physiological stress and a greater demand on bodily reserves. The immune system is particularly affected by the process of aging [1], especially regarding the numbers and functions of T lymphocytes. Studies of older humans and animals have revealed that the most noticeably altered T cell functions are the production of IL-2 and the clonal expansion of effector and memory T cells [2]. These functions are the outcome of the activation of T cells and it can be hypothesized that defects or alterations in the proximal events of T cell activation will strongly affect the efficiency of the immune response. In other words, appropriate signal transduction cascades trigger a response, but changes in the early events of T cell signaling will result in less effective, altered overall responses [3,4]. The antigen-presenting cell (APC)-dependent major histocompatibility-restricted activation of T cells requires an intimate communication between APC and T cells by way of the formation of an immune synapse [5,6]. It is now generally accepted that the formation of the immune synapse could play an important role in the assembly of the complex machinery of T cell signal transduction which occurs in specialized microclusters of the plasma membrane of the cell [5,7]. These microdomains are resistant to detergent solubilization at 4°C and have been designated “detergent-resistant plasma membrane domains” or “lipid rafts” (LR) [8]. This article will discuss the alterations in T cell signaling with aging in relation to LR functions and the role of cholesterol in this phenomenon.

## What is Immunosenescence?

As life expectancy of the populations of industrialized nations continues to increase, the emergence of many diseases associated with aging has become a major medical and economic concern. For instance, the incidence of infections, cancers and chronic inflammatory diseases such as atherosclerosis and neurodegenerative diseases which increase with age has become an important human and cost-related problem [9]. Although the underlying causes relating aging to increased pathologies is not fully understood, it is recognized that alterations of the immune system play an important role [10]. The immune system is a complex interactive system composed of many different cells and cell subpopulations that are altered with age, but not all to the same extent, and thus do not contribute equally to immunosenescence. This should be conceptualized as dysregulation of a system constantly trying to adapt and to maintain homeostasis in the face of inputs and outputs which are still only crudely defined [11]. Although numerous studies on age-related immune alterations have been published, the conclusions remain controversial due to differences including those between species and the lack of a definition of physiological aging. Furthermore, the exclusion of some latent disease states, nutritional, genetic and environmental differences add to the complexity of understanding the mechanisms of the physiological changes and their overall contributions to aging [12]. However, the direct clinical consequences of decreased immune responses with aging seem quite clear. These are revealed by the increased incidence and severity of infections, cancers and autoimmune disorders [10,13] and can be related to the hallmark of immunosenescence which is altered T cell function with aging [14]. Additional changes in immune system components have been described, but they are much less marked and may often be secondary to changes in T cells. The changes include not only the T cell-dependent B cell responses, but also innate components sensitive to T cell feedback, especially APC [15]. Although age-related changes in the immune response are multifactorial, it is reasonable to assume that an alteration in T lymphocyte activation is a central issue in age-related modifications of the immune response.

### Changes in T lymphocyte activation with aging

#### T cell signaling *via* T cell receptor and CD28

**Figure 1:**
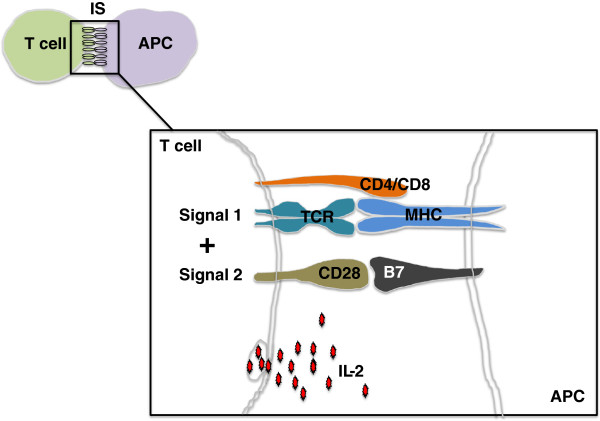
**Signal 1 and 2 during T cell activation.** Upon antigen presentation by professional antigen presenting cells (APC), multiple stimulatory molecules control full activation of T cells. Signal 1 is delivered by CD4/CD8 and T cell receptor (TCR)/major histocompatibility (MHC) interaction while signal 2 is delivered by CD28/B7 interaction (or other co-stimulator-receptor pairs) The two signals together are required to induce IL-2 production which will have autocrine and paracrine effects.

T cells are activated by antigen presented by APC [16]. The most efficient APC are dendritic cells, although monocytes/macrophages and B cells are also able to present antigens. APC presentation within the context of major histocompatibility restriction triggers a cascade of signaling events in naive T cells that culminates in the activation of a set of genes resulting in the synthesis and secretion of IL-2 and many other cytokines and factors (Figure 1). In addition, activated T cells initiate immune responses and traverse differentiation pathways resulting in the development of different T cell subsets, according to the priming conditions [17] regulated by the cytokines and chemokines secreted in the immediate vicinity [7]. A universal requirement for any T cell response is marked proliferation to accomplish clonal expansion that is critical for generating T cells in sufficient numbers to eliminate the antigen. Memory T cells possess different stimulatory requirements than naïve T cells, but the efficiency of both naïve and memory cell responses is diminished with age.

The primary receptor for T cell activation is the T cell receptor (TCR) which delivers “signal 1”. However, this is not sufficient for full T cell activation and, to avoid anergy, T cells must receive a second signal delivered by a plethora of co-receptors [17]. A number of these have been described [16] but, functionally, the most important of these seems to be CD28 which, by recognizing CD80 and CD86 ligands on APC, delivers “signal 2” (Figure 1) that is essential to sustain T cell activation [18]. In addition to its activity as a co-stimulatory molecule, CD28 is involved in re-expression of the TCR, recruitment and stabilization of T cell LR to the immune synapse and the activation of plasma membrane lipid metabolism through upregulation of the PI-3 K signaling pathway [19,20]. Furthermore, CD28 co-stimulation synergizes with TCR activation and induces IL-2, IL-4, IL-5, TNF and granulocyte-macrophage colony stimulating factor (GM-CSF) production *via* NF-kB activation [19,21]. More co-stimulation is required for the activation of naïve cells than for memory cells. It is now well established that CD28 expression is decreased with aging in CD8^+^ and CD4^+^ lymphocytes with a bias towards a greater effect on CD8^+^ T cells [22].

#### Age-related alterations in T cell signaling

There are several age-associated alterations in these T cell activation pathways, as observed in experimental animals [23,24] and humans [2]. The most important changes occur in CD4^+^ T cells resulting in the decreased production of IL-2 and clonal expansion [3]. Although there are no changes in TCR number at the cell surface, the number of CD28 co-stimulatory molecules decreases with aging [25]. Due to the essential role of CD28 to prevent T cell anergy, the decrease in number of this receptor could affect T cell responses in aged humans. Nearly all the activity of the signaling pathways associated with TCR activation or IL-2 receptor response have been found to be altered with aging [26]. There is an alteration in the early steps of T cell activation including protein tyrosine phosphorylation, calcium mobilization and the translocation of protein kinase C to the plasma membrane. In addition, subsequent steps of the signaling pathways including the Raf-Ras-MAP kinase pathway are impaired. The decline in proximal and intermediate events of transmembrane signaling leads to the decreased activity of transcription factors, especially NF-kB and NF-AT [27] and results in altered production of cytokines with aging in T cells. This has been observed in the case of T helper-1-dependent cytokines such as IL-2, IL-6 and TNF-α production [28].

#### *Lck regulation loop alterations with aging*

**Figure 2:**
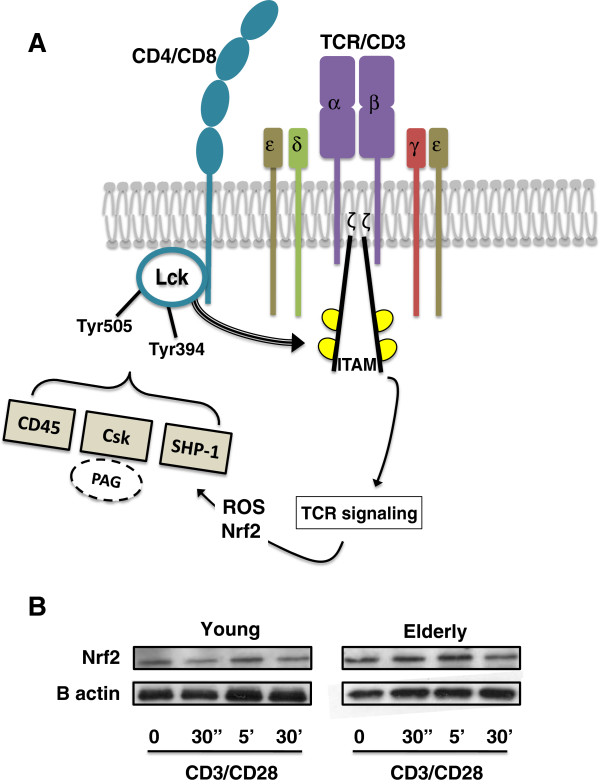
**Lck is a central node in the T cell receptor signaling pathway.** (**A**) Following T cell receptor (TCR)/CD3 complex ligation, Lck phosphorylation sites (inhibitory Tyr505 and activatory Tyr394) regulate Lck activity. The phosphorylation of immunotyrosine-based activating motifs (ITAMs) by Lck induces recruitment of other kinases and leads to T cell activation. Phosphorylation of Lck is regulated positively by CD45 and negatively by C-terminal Src kinase (Csk), itself regulated by phosphoprotein associated with GEMs (PAG), and *Src* homology 2 domain-containing protein tyrosine-phosphatase 1 (SHP-1). The regulation of phosphatases such as SHP-1 is strongly influenced by oxidative stress (reactive oxygen species) and the Nrf2 pathway. (**B**) Nrf2 quantification in resting and activated (anti-CD3/CD28) T cell cytoplasm in young versus older individuals is shown (a representative blot is shown). In addition, there are other regulatory mechanisms that function rapidly as negative feedback loops from the TCR signalosome itself [19]. One of these involves phosphatases, especially SHP-1 [35]. Reduction of SHP-1 activity upon stimulation lowers the threshold for TCR activation. It seems that under weak stimulation activated Lck associates with and phosphorylates SHP-1, the activity of which then becomes reduced, but not sufficiently to allow full signaling to occur. Thus, under strong stimulation, Lck activity would increase or become sufficient when SHP-1 activity is reduced very early in the signaling cascade. In turn, when the signal weakens, SHP-1 activity increases and consequently downregulates Lck activity [36]. Many other negative feedback mechanisms are in operation during the early and late phases of TCR signaling [19].

Lately it became evident that one of the most important changes underlying altered TCR signaling is at the level of the Lck pathway (Figure 2A). Lck is at center stage of TCR signaling initiation [29]. Phosphorylation of the TCR by Lck initiates downstream signaling by creating binding sites that recruit the cytosolic kinase ZAP70 (Zeta-chain-associated protein kinase 70) to the cell membrane [30]. Lck is regulated by the C-terminal Src kinase (Csk) by transphosphorylation and by dephosphorylation by phosphatases. Lck cycles between an unphosphorylated (primed state), active and inactive states. The Lck C-terminal tyrosine Tyr505 is phosphorylated by Csk which allows the Lck C-terminus to bind its own SH2 domain, which closes the molecule. In contrast, the trans-autophosphorylation of Lck activation loop Tyr394 activates the kinase activity *via* rearrangements of the active site [31]. In resting cells equilibrium probably exists between the Lck phosphorylated on various Tyr sites resulting in various levels of kinase activity determined by the amount of Csk, CD45 and SHP-1 (*Src* homology 2 domain-containing protein tyrosine-phosphatase 1) activities (Figure 2A). Reduced phosphatase activity within a close contact zone of Lck results in Lck activation by trans-autophosphorylation of Tyr394 [32]. However, it seems that some part of Lck is in an active form in resting T cells where a decrease of phosphatase activity will induce the signaling cascade as well as segregation in various membrane domains. Indeed, at basal states, Lck partitions preferentially into LR compartments where active and inactive Lck forms co-exist at different stoichiometry and interact with distinct pools of CD4 and Csk [33]. Thus, modulation of phosphatase activities in the close contact zone is an essential stage of TCR activation to release the already active Lck. Furthermore, in resting lymphocytes, Fyn has been shown to be the predominant Src-family tyrosine kinases (SFK) responsible for PAG/Cbp (phosphoprotein associated with GEMs/Csk-binding protein) phosphorylation recruiting Csk to LR to regulate Lck activity [34]. Consistent with this, strong TCR stimulation reduces constitutive PAG phosphorylation, thus favoring the dissociation of Csk, the main negative regulator of Lck activity. This is believed to induce TCR signal transduction.

#### *Nrf2 activation alteration with aging*

Aging is characterized by an increased level of oxidative stress. This also influences T cell activation. The mechanism of redox-dependent regulation of signaling pathways implies that receptor-mediated generation of reactive oxygen species (ROS) influences the balance between protein tyrosine kinase and phosphatase activities. Protein tyrosine kinases and phosphatases are redox-sensitive targets with an oxidation-sensitive active site cystein [37]. During receptor stimulation the transient increase of ROS may inactivate SHP-1 and thus facilitate TCR signaling (Figure 2A). However, in a setting where there is a constant increase in ROS, as in aging, SHP-1 regulation is altered and a negative regulatory role is favored in both the resting state and under stimulation. Another important constituent of the activation process is the cap ‘n’ collar basic region leucine zipper family member nuclear factor erythroid 2-related factor − 2 (Nrf2), a transcription factor [37]. Nrf2 is widely expressed in different cells and tissues, and is activated by oxidative and electrophilic stimulating agents, including ROS. Nrf2 modulates antioxidant gene expression by interacting with antioxidant response element. Nrf2 is tethered to the suppressor protein Kelch-like ECH-associated protein 1 in the cytoplasm under conditions of low oxidative stress, and is degraded by the ubiquitin-proteasome pathway. Under oxidative stress, the complex dissociates, and Nrf2 translocates to the nucleus and enhances the expression of Nrf2/antioxidant response element-associated antioxidant genes [38]. Our own preliminary results suggest that Nrf2 is decreased upon stimulation of T cells from young individuals, indicating an increase of oxidative stress, while in the older people no changes in the Nrf2 levels were detected. This suggests a constantly increased ROS level, which is probably compensated for by alternative mechanisms inhibiting the translocation of Nrf2 into the nucleus (Figure 2B). This results in altered antioxidant generation, as has been demonstrated in T cells by the decrease of reduced glutathione and increase of oxidized glutathione levels [39]. Thus, with aging, increased oxidative stress together with the changes of tyrosine kinase and phosphatase activities, and their altered localization in the LR, contribute to altered T cell signaling with aging.

#### Role of lipid rafts in T cell signaling

Studies in model lipid bilayers have established that they can exist in an ordered (l_o_) gel-like phase and a disordered (l_d_) phase. In the case of the cell plasma membrane, the presence of cholesterol that associates with sphingolipids and other lipids allows the formation of microdomains in an l_o_-like fluid phase that floats within the rest of the lipid bilayer, which is in the l_d_ phase. The term “lipid rafts” (LR) has been coined to designate these (l_o_) microdomains [40,41]. LR are dynamic structures that are generally thought to be small, submicroscopic regions depleted of unsaturated phospholipids but enriched in cholesterol, alkyl chain-saturated sphingolipids and some lipid-anchored proteins [42]. Of significance, studies with lymphocytes have revealed that LR allow the recruitment of signaling proteins [43,44]. The coalescence of LR, dubbed “lipid rafting”, is essential for the formation of the immune synapse and signal transduction in T cells [45]. However, this view was challenged by data from new imaging techniques such that conceptualization of the nature and function of LR is evolving [46-48]. Thus, LR are now considered as actual structures occupying spaces in the membrane as fluctuating dynamic nanoscale assemblies of sphingolipids, cholesterol and proteins that can be stabilized into platforms important for signaling and membrane trafficking. They can be further clustered into a second state by specific lipid-lipid, protein-lipid and protein-protein interactions [48] to generate more stable, selective and functional platforms. The merger of specific nanoscale rafts into larger and more stable platforms represents the functionalization of specific rafts in membrane trafficking as well as in signal transduction. For these events to occur the protein and lipid composition needs to be strictly fine tuned.

TCR are activated in microclusters subsequently transported to the locus of APC contact by actin filaments, generating a so-called central supramolecular activation cluster in which many TCRs are already dephosphorylated and inactive [49]. These data questioned the role of immune synapses in the signal transduction process, but helped to establish different roles for their components. This also helped to reconceptualize the composition and role of LR. Since then, modern high resolution imaging revealed the existence of complexes with up to 7 to 20 TCRs, manifested as protein islands 70 to 140 nm in diameter [50]. Were the resting states of raft proteins associated with nanoscale assemblies of raft lipids (for example, sphingolipids and cholesterol), such variances in size would result from the ability of these dynamic structures to coalesce. Stabilization of fluctuating nanoscale structures could facilitate increased access to Lck to mediate TCR signaling [33,51]. In this setting, how proteins and lipids (mainly cholesterol) interact remains an important question. How does the lipid context around the TCRs change the inactive to the activated state?

#### Role of cholesterol

The role of LR in T cell signaling has been challenged recently [47,48] despite substantial evidence in favor of their involvement in this process [46]. Several studies concluded that LR are indeed involved in T cell signaling because of the effects of acute cholesterol depletion - for example, both aggregation of plasma membrane LR and an increase in their numbers in the plasma membrane were noted on partial cholesterol depletion. This preceded Src family kinase activation, leading to increased co-localization between signaling molecules and resulting in an early T cell signaling response accompanied by marked actin polymerization in the plasma membrane [52]. It was also found that the kinase inhibitor PP2 did not prevent LR aggregation, which is in line with microcluster formation preceding T cell signaling [53]. The quantity of cholesterol extracted from the membrane can have differential effects on signal transduction, emphasizing that the lipid-protein interaction is as important as the protein-protein interaction in the signaling process.

**Figure 3:**
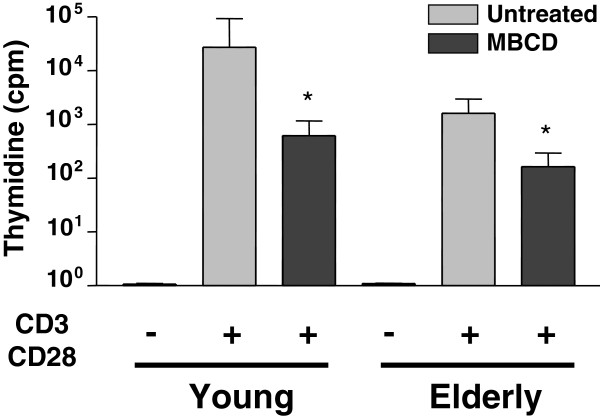
**Impact of lipid factors on T cell function.** Proliferation was measured after T cell activation with CD3/CD28 antibodies. Peripheral blood mononuclear cells (PBMC) from young and older people were treated with 5 mM methyl β-cyclodextrin (MBCD) for 30 minutes to extract membrane cholesterol prior to culturing. *P <0.01.

Cholesterol is known to increase the thickness of lipid bilayers, as well as their stiffness. Both of these parameters are very important for interaction with membrane proteins. A series of investigations in the late 1980s had already suggested that biochemical and biophysical alterations at the level of the cell plasma membrane could be associated with the altered immune response with aging in mice [54,55]. It was noted that there were modifications with aging in the composition of the lymphocyte cell plasma membrane lipids as well as its fluidity. Earlier data from our laboratories have shown that the levels of plasma membrane cholesterol, the major stabilizing component of LR, were increased two-fold in T lymphocytes of older individuals, resulting in a plasma membrane that was less fluid than in T cells from young individuals [26]. We hypothesized that these observations had bearing on the events of T cell activation with aging. We investigated whether there were alterations in the proximal events of T cell activation upstream of the signaling cascade or at the very early events of signaling. We had used HPLC, thin layer chromatography and other lipid detecting techniques to show that there were marked changes in plasma membrane composition (fatty acids, phospholipids) in T cells of middle-aged normolipemic subjects [56]. We put forward the hypothesis that these changes could be related to the decreased T cell functions associated with immunosenescence. We attempted to modulate the levels of plasma membrane cholesterol in T cells from older subjects *in vitro* to determine whether this manipulation re-established efficient T cell signaling/functions. Initial experiments using the cholesterol sequestrating compound methyl β-cyclodextrin (MBCD) did not yield results expected on the basis of the hypothesis mentioned above. It was observed that, besides decreasing plasma membrane cholesterol, MBCD had an independent signal-disrupting activity. We therefore also used inhibitors of cholesterol synthesis (lovastatin) in Jurkat T cells. In this case, significantly reduced cell growth and induced apoptosis at high concentrations were observed, in agreement with the fact that only a few studies have demonstrated the immunomodulatory role of statins in human T cells [57]. Reciprocally, we attempted to “age” T cells of young subjects by increasing the cholesterol content to the levels found in T cells of older subjects. This led to a decreased proliferative response as well as IL-2 secretion, suggesting that homeostatic concentrations of plasma membrane cholesterol are important for T cell activation (Figure 3). An attractive approach *in vivo* might therefore be to use high-density lipoprotein (HDL) as a physiologic means to extract cholesterol by the so-called reverse transport cholesterol mechanism. HDL particles or ApoA-I interact with cells to promote the efflux of cholesterol *via* binding to specific transporters of the ATP-binding cassette (ABC) gene family [58]. ABCG1 induces cholesterol efflux to HDL particles [59]. HDL preferentially interacts with LR and depletes cholesterol from these microdomains [60]. By this extraction effect, HDL may modulate the signaling and the functions of T cells. More work is needed to determine the exact role of cholesterol and HDL in the altered activation of T cells with aging.

There are no well documented molecular mechanisms to account for increased plasma membrane cholesterol content in T cells of normolipemic older subjects. However, a dysregulation of cholesterol uptake involving the low-density lipoprotein receptor, an increase in the synthesis of cholesterol resulting from upregulation of HMG-CoA reductase activity, an alteration in the activity of the ACAT enzyme or a deficiency in HDL-dependent reverse cholesterol transport are all possible causes. Further studies of cholesterol metabolism in T cells and the modulation of the cholesterol content of the plasma membrane are needed to shed light on the alterations and the functional changes of LR and the early events of T cell activation with aging.

#### Alterations in lipid raft functions with aging

LR move across the plasma membrane lipid bilayer as discrete complexes to assemble the signalosome [61] prior or concomitantly to the formation of the immune synapse. LR clustering may depend on GM1 (monosialotetrahexosylganglioside), GM3 and flotillin-1 [62] as well as reorganization of the cytoskeleton. Whereas some of the signaling components are constitutive parts of LR (Lck, Cbp/PAG), others are recruited during activation (TCR, CD28, IL-2 receptor, LAT, PI-3 K). We were the first group to report a study of LR alterations in older humans with aging [63]. We have shown that LR are distributed homogeneously in T cells of young subjects, whereas we observed a different LR pattern and coalescence in the quiescent state in T cells of older subjects [26]. These observations may reflect, to some extent, low-grade activation with aging as part of the “Inflamm-Aging” model [64]. We have also reported an alteration in the function of LR with aging. Using confocal microscopy, we have shown that LR coalescence is altered with aging in T cells activated by a combination of anti-TCR and anti-CD28 antibodies. LR poorly coalesced in CD4^+^ T cells of older subjects [22] although the alterations were less pronounced in the case of CD8^+^ T cells. Furthermore, we have reported alterations in the recruitment and activation of Lck and LAT into LR of T cells from older humans [26]. One important finding was that CD28 and the IL-2 receptor were weakly recruited to LR in CD4^+^ T cells of older subjects. In contrast, these proteins were already located in LR in CD8^+^ T cells from older subjects prior to stimulation. These observations suggested that the assembly of the signaling machinery in CD4^+^ T cells relies largely on LR, whereas a pre-assembly of the signalosome may be present in CD8^+^ T lymphocytes, as proposed by us [22] and by others [65,66]. Currently we do not know why this alteration of the plasma membrane occurs with aging. An intrinsic alteration of cholesterol metabolism in older subjects could contribute to these lipid changes [67]. Therefore, an alteration in the plasma membrane composition of T cells with aging seems to play a fundamental role in their decreased functions as observed in immunosenescence. Moreover, an alteration of the cytoskeleton in T cells with aging can further contribute to altered signaling *via* altered LR functions.

## Conclusions

Increasing numbers of older individuals not only in industrialized nations represents major social and medical challenges. Concomitantly, the incidence of age-related diseases, many of which are influenced by the dysregulation of the immune system, is increasing. There is a large corpus of experimental data suggesting that the immune response, mainly the T cell response, is dysregulated in aging. The causes of this dysregulation remain largely unknown, although evidence for alterations in T cell receptor and CD28 co-receptor signaling is widely accepted. One unifying cause for these alterations could involve the initial events of signal transduction and the role of the plasma membrane. The novel concept that the plasma membrane is composed of privileged signal transduction microdomains (LR) that create a functional dynamic environment to assemble the signalosome and the constitution of the immune synapse provides a working framework to address the molecular mechanisms of immunosenescence. Changes in the lipid composition of the plasma membrane, especially in LR, can be envisaged as a key event that adversely affects T lymphocyte signaling in older subjects. These functional changes may have serious clinical consequences such as increased infections, cancers, autoimmune diseases and thus poor quality of life. According to one estimate, more than 50 pathologies have been associated with dysfunctional LR [68]. The inter-individual susceptibility to the diseases of aging may be the cause or the consequence of the differential changes in the immune response (which is associated with T cell pool composition). It is of fundamental importance to understand the role of LR to be able to intervene more precisely during the evolution of diseases affecting older people. New imaging techniques should be applied to the study of signaling events in T cells of healthy older subjects [46,47]. These studies could lead to the elaboration of new intervention tools in the management of the physiological as well as the pathological process of aging.

Data from our laboratories and others have provided a basis to pursue studies to understand the molecular mechanisms of T cell signaling deficiencies associated with aging, the involvement of LR, the formation of the immune synapse and the assembly of the signaling machinery. Moreover, investigating T lymphocyte cholesterol metabolism will help to understand the increase of cholesterol content in the membrane. The role of free radicals should also be assessed in connection with antioxidant defense. Thus, a better understanding of immunosenescence by intensive research and the development of new methods and strategies to intervene in its evolution are essential for improving the quality of life of an increasingly larger older population.

## Abbreviations

ABC: ATP-binding cassette; APC: antigen-presenting cell; Cbp: Csk-binding protein; Csk: C-terminal Src kinase; HDL: high-density lipoprotein; HPLC: high performance liquid chromatography; IL: interleukin; LR: lipid rafts; MBCD: methyl β-cyclodextrin; Nrf2: nuclear factor erythroid 2-related factor-2; PAG: phosphoprotein associated with GEMs; ROS: reactive oxygen species; SHP-1: *Src* homology 2 domain-containing protein tyrosine-phosphatase 1; TCR: T cell receptor; TNF: tumor necrosis factor; ZAP70: Zeta-chain-associated protein kinase 70.

## Competing interests

The authors declare that they have no competing interests.

## Authors’ contributions

All authors contributed to writing the manuscript. All authors read and approved the final manuscript.
